# Jurassic Palynology from “The Dinosaur Coast” of Asturias (Lastres Fm., Northwestern Spain): Palynostratigraphical and Palaeoecological Insights

**DOI:** 10.3390/biology11121695

**Published:** 2022-11-24

**Authors:** Artai A. Santos, Laura Piñuela, Iván Rodríguez-Barreiro, José Carlos García-Ramos, José B. Diez

**Affiliations:** 1 Centro de Investigación Mariña, Universidade de Vigo (CIM-UVIGO), 36310 Vigo, Spain; 2 Departamento de Xeociencias Mariñas e O.T., Universidade de Vigo, 36310 Vigo, Spain; 3 MUJA—Museo del Jurásico de Asturias, Rasa de San Telmo, 33328 Colunga, Spain

**Keywords:** palynology, plant communities, wildfires, Late Jurassic, “The Dinosaur Coast”, North Spain

## Abstract

**Simple Summary:**

The Upper Jurassic deposits of the Lastres Formation crop out on the Asturian coast (northwest of the Iberian Peninsula), in the so-called “The Dinosaur Coast”. This formation presents a high abundance of dinosaur remains and other vertebrates. Despite the deep knowledge about its fauna and environment, practically nothing is known about the plant communities that formed the landscape of the region at the end of the Jurassic. We present here the first palynological data of the Lastres Fm., identifying a rich and abundant palynological assemblage formed by more than 60 different taxa. The presence of some taxa with biostratigraphic value suggests a Kimmeridgian-Tithonian age for this formation. On the other hand, the botanical affinities of the taxa found indicate that the vegetation of the “The Dinosaur Coast” of Asturias would not be homogeneous, but would be formed by a mosaic of different plant communities that would adapt to the variety of environments present in the region. The presence of forest areas probably represented a protected environment as well as a food source for herbivorous dinosaurs. Analysis of charcoalified wood remains suggests that palaeofires were relatively recurrent in the study area.

**Abstract:**

Abundant fossils of vertebrates (mainly footprints and bones of dinosaurs) and numerous invertebrates occur in the Upper Jurassic deposits of the Lastres Formation in the Asturias region, North of Spain. However, no palynological study has been published from this geological formation; therefore, much palaeoenvironmental and palaeoecological information is still unknown. In this study, a total of 62 morphospecies, belonging to 49 different morphogenera were identified, including pollen, spores, algae remains, fungi spores, dinoflagellates, foraminifera, and scolecodonts from four different locations on the Asturian coast. Spores are the dominant group of palynomorphs, both in diversity and abundance, contrasting with the minor diversity of pollen grains. The age of some key taxa indicates that the palynological assemblage cannot be older than the Kimmeridgian, suggesting a Kimmeridgian-Tithonian age. The botanical and environmental affinities of the pollen and spores indicate the presence of different plant assemblages, including plant communities from humid areas such as the margin of rivers and small freshwater ponds that were dominated by bryophytes and ferns, and a coastal plant community that would inhabit arid areas and would be dominated by gymnosperms and some pteridophytes. The SEM analyses of wood remains show the abundance of charcoalified remains suggesting that wildfires were usual in “The Dinosaur Coast” of Asturias during the Kimmeridgian.

## 1. Introduction

“The Dinosaur Coast” of Asturias in the Northwest of Spain extends over several kilometers of Jurassic deltaic deposits exposed along the Asturian coast. Its Upper Jurassic deposits, specifically those from the Lastres Formation, are a benchmark in the study of the coastal fauna of this period of time and an important touristic attraction in the region. The Lastres Formation has been widely studied both sedimentologically [1,2,3,4,5] and palaeontologically [6,7,8,9,10,11,12,13]. The Lastres Formation yields a rich, diverse, and abundant faunal assemblage. The fossils published so far include different groups of dinosaurs [10,11,12,13] but other vertebrate remains and tracks have been found, such as turtles [8], crocodylomorphs [3,9], or pterosaurs [13]. In addition, these deposits are also rich in invertebrates such as ammonites [6,14,15], bivalves [16], or ostracods [7,17].

This diversity of fossils of animals that characterizes the Lastres Formation contrasts with the very scarce knowledge about their plant communities. Few studies concerning palaeobotany of this formation have been published, including wood remains of conifers attributed to *Protocupressinoxylon* sp. [2] and to *Protocupressinoxylon purbeckensis* [18]. So far, no palynological studies have been published on the Lastres Formation.

Although the Lastres Formation is usually referred to as Kimmeridgian, its age is still controversial. Some biostratigraphical data have suggested an early Kimmeridgian age for the upper part of the Lastres Formation based on ammonites [6,14,15], but other authors considered a late Kimmeridgian age for the lower and middle part and a Tithonian age for the upper part of the formation based on both ammonites and ostracods [5,7,17]. Consequently, to solve this stratigraphic conundrum, it is necessary to provide new biostratigraphical data; in this context, palynostratigraphy could be a useful tool.

The references to Jurassic palynological studies from the Iberian Peninsula are scarce and mostly focused on Lower and Middle Jurassic deposits. In Portugal there are some studies from the Algarve Basin in Pliensbachian to Kimmeridgian outcrops [19,20,21], and from the Lusitanian Basin in Pliensbachian to Bajocian marine stratigraphic levels [22], in marine Pliensbachian-Toarcian deposits [23,24,25] and from the Callovian-Oxfordian boundary [26]. In addition, there are other studies focused on Upper Jurassic deposits from the Oxfordian of Leiria [27], the Oxfordian-Kimmeridgian of Porto de Mos [19], and from the Upper Oxfordian to Tithonian of the Lusitanian Basin [28].

In Spain, there are only a few palynological studies from Upper Jurassic deposits, including the palynology from the Bathonian-Oxfordian stratigraphic levels in the Iberian Range [20,29,30] and the Lower Kimmeridgian deposits from Jaen and Teruel provinces [31]. Recently, some palynological studies were focused on the J/K boundary in the Galve area from Teruel [32], and the Cameros Basin in Burgos [33]. More specifically, in the studied area, few palaeobotanical studies were performed: a preliminary palynological study of the Vega Fm. [34] and a recent macroflora discovery in the Lastres Fm. [35].

This scarcity of palynological studies in the studied area, especially for Upper Jurassic deposits, raises many questions about the evolution and distribution of plant communities in the Iberian Peninsula during the Late Jurassic. Our work represents the first palynological data from the Lastres Fm. of special interest due to its vertebrate fossil abundance, also providing new data on the knowledge of the plant communities of this period. Therefore, the main objectives of this work are: (1) to characterize the palynological assemblage from the Lastres Fm.; (2) to provide new biostratigraphic data improving the chronostratigraphic resolution of the formation; and (3) to discuss the palaeoecological and palaeoenvironmental implications of this new palynological assemblage.

## 2. Geographical and Geological Context

**Figure 1:**
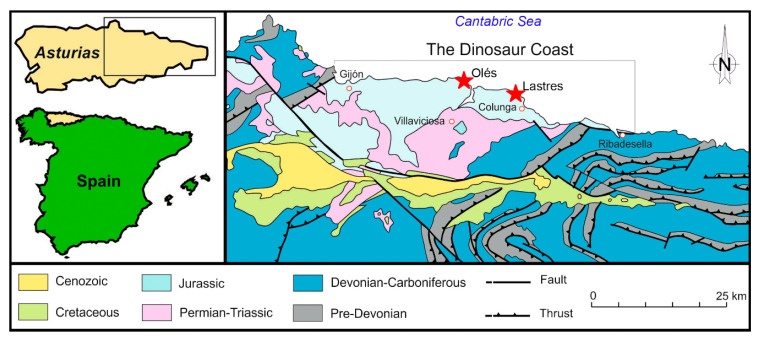
Geological and geographical map from the Asturias coast. The studied locations (Oles and Lastres) are indicated with red stars.

**Figure 2:**
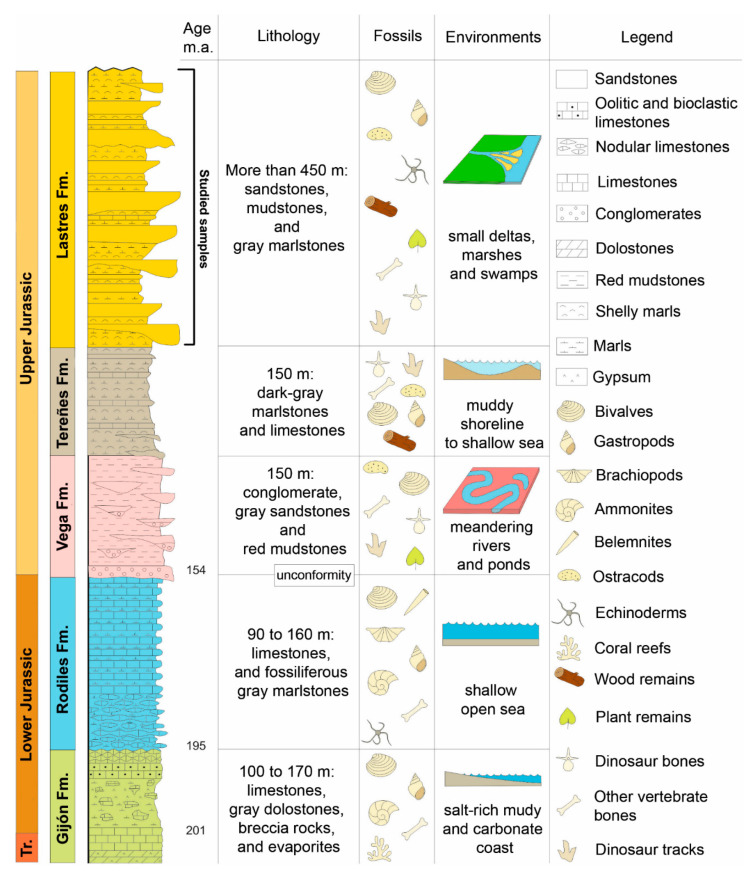
Lithostratigraphical and palaeoenvironmental context of “The Dinosaur Coast” of Asturias.

The Upper Jurassic sedimentary rocks of Asturias crop out along the coast between Gijón and Ribadesella (Figure 1 and Figure 2). The succession is more than 600 m thick, and consists of three lithostratigraphic units, which yielded an important sample of dinosaur footprints and skeletal remains (Vega, Tereñes, and Lastres formations, see Figure 2).

The Lastres Formation represents a splendid Jurassic example of a fluvial-dominated lagoonal delta sourced mostly by high-sinuosity rivers. It is characterized by alternating grey sandstones, mudstones, marls and occasional conglomerate levels [3,36]. Although the main environments of the delta system are well represented along the formation, all the selected samples with palynomorphs are included mostly in the delta plain and locally in the delta abandonment facies.

**Figure 3:**
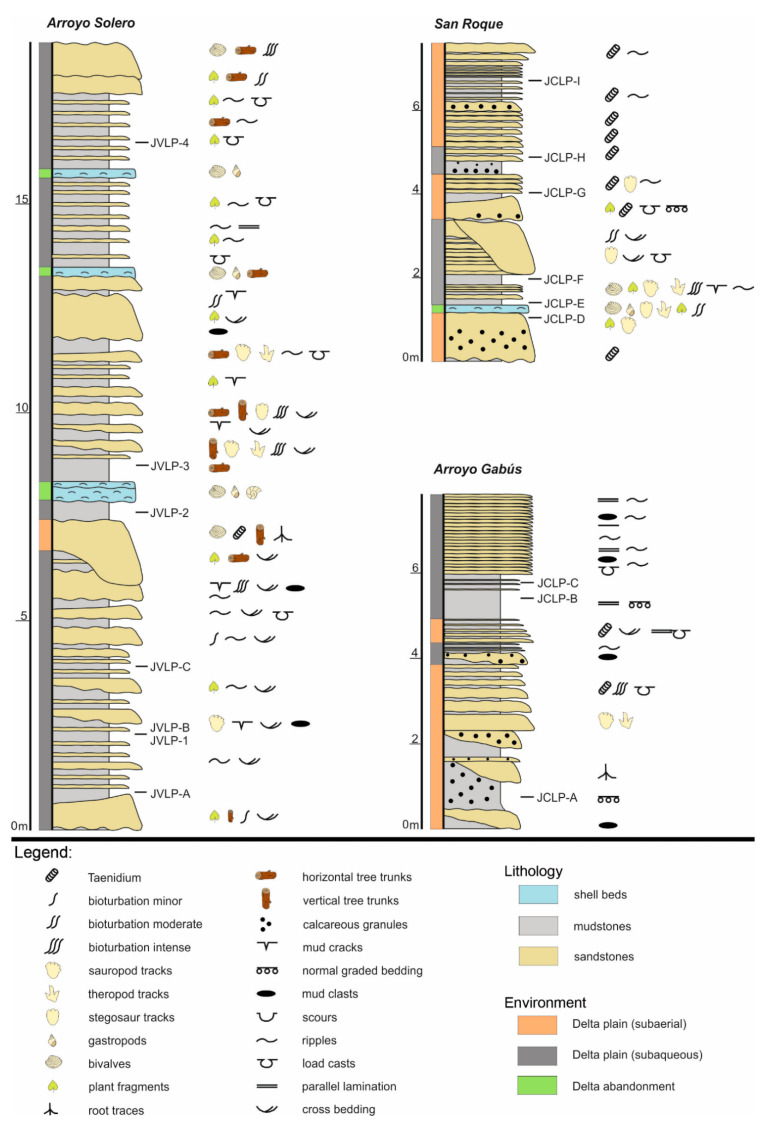
Stratigraphic sections of the Lastres Formation indicating the locations of the samples.

The apparent absence of tides in this restricted lagoonal delta prevents the distinction between the upper and lower delta plain but it is possible to differentiate between a subaerial well-drained delta plain and a subaqueous poorly drained delta plain (Figure 3).

The siliciclastic sedimentation was repeatedly interrupted by carbonate shell beds, representing the typical delta abandonment facies. These last facies (shell beds) formed during transgressive periods driven by regional or local increases in tectonic subsidence (normal faults related to contemporary rifting processes), eustatic sea-level rise or lessening/stopping in sediment supply (i.e., avulsion processes). During the Kimmeridgian-Tithonian, the Iberian Peninsula was a big island between the Euroasiatic, African and American Plates.

## 3. Material and Methods

For this work, three different sections from the Lastres Fm. were selected according to the ones that are more complete sedimentologically and chronologically: Arroyo Solero in Oles (Villaviciosa, Asturias) and Arroyo Gabús and San Roque, both in Lastres (Colunga, Asturias). For the palynological study, the samples were taken from levels with fine-grain sediment and rich in organic matter. A total of 16 palynological samples were collected, seven from Arroyo Solero (JVLP1, JVLP2, JVLP3, JVLP4, JVLPA, JVLPB, and JVLPC), three from Arroyo Gabús (JCLPA, JCLPB, JCLPC), and six from San Roque (JCLPD, JCLPE, JCLPF, JCLPG, JCLPH, and JCLPI).

**Figure 4:**
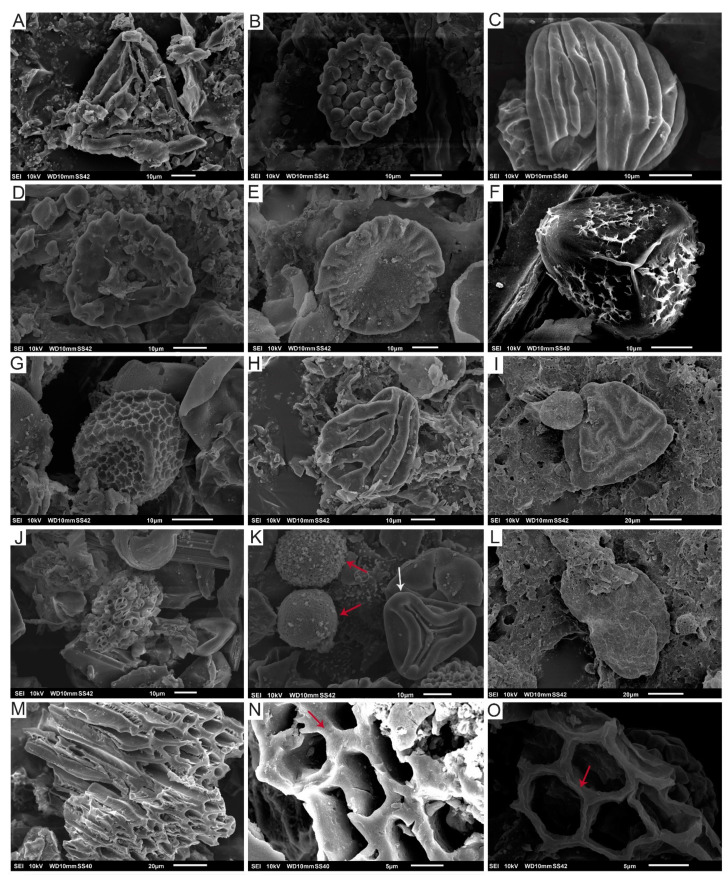
Palynomorphs from the Lastres Fm. under SEM: (**A**) *Cicatricosisporites* sp., SEM-V1b-Lastres; (**B**) *Leptolepidites* sp., SEM-VA-Lastres; (**C**) *Contignisporites cooksonii*, SEM-V1-Lastres; (**D**) *Klukisporites* sp., SEM-CA4-Lastres; (**E**) *Callialasporites dampieri*, SEM-V1-Lastres; (**F**) cf. *Camarazonosporites rudis*, SEM-V1b-Lastres; (**G**) *Retitriletes clavatoides*, SEM-V1b-Lastres; (**H**) *Striatella balmei*, SEM-V3-Lastres; (**I**) *Striatella scanica*, SEM-CAb-Lastres; (**J**) *Botryococcus* sp., SEM-V1-Lastres; (**K**) *Dictyophyllidites harrisii* (white arrow); pirite (red arrows)*,* SEM-V1-Lastres; (**L**) *Pinuspollenites* sp., SEM-CA3-Lastres; (**M**) Remains of wood with almost totally fused cell walls, SEM-V1-Lastres; (**N**) Detail of fused cell walls in wood (see red arrow), SEM-V1b-Lastres; (**O**) Unfused cell walls in wood (see red arrow), SEM-VB-Lastres.

**Figure 5:**
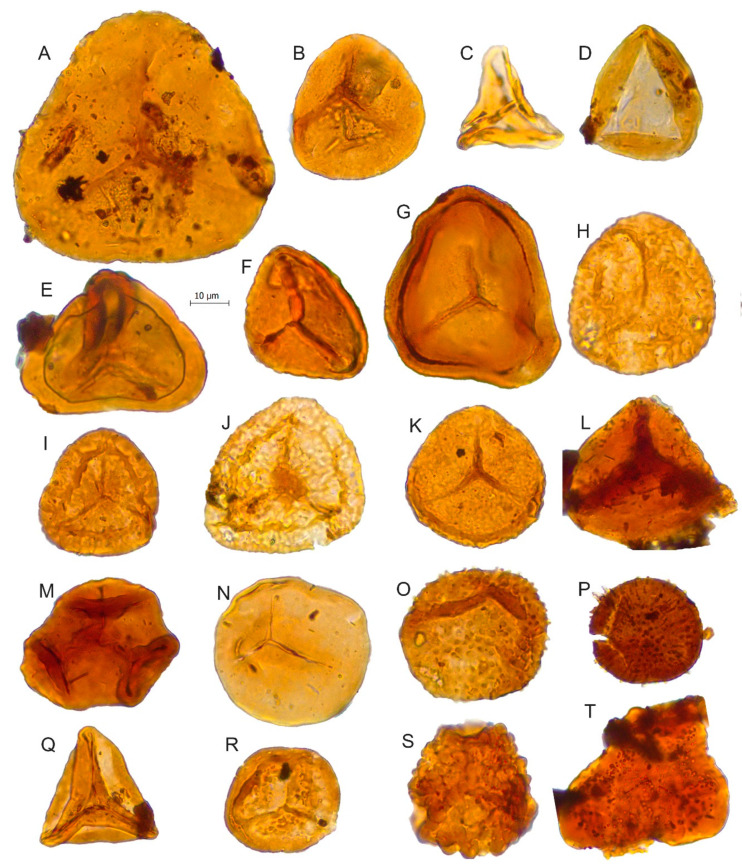
Scale bar: 10 µm. (**A**) *Cyathidites australis*, JVLP-2-4-H160; (**B**) *Cyathidites minor*, JCLP-B-4-O270; (**C**) *Gleicheniidites senonicus,* JVLP-C-2-T372; (**D**) *Deltoidospora* sp., JVLP-A-1-J054; (**E**) *Murospora* sp., JVLP-B-2-C281; (**F**) *Matonisporites equiexinus*, JVLP-3-1-D510; (**G**) *Matonisporites* sp., JVLP-3-1-Q430; (**H**) cf. *Camarazonosporites rudis*, JVLP-3-1-J360; (**I**) *Staplinisporites caminus*, JVLP-3-1-G440; (**J**) *Staplinisporites* sp., JVLP-C-2-R280; (**K**) *Stereisporites antiquasporites*, JVLP-1-2-N470; (**L**) *Biretisporites potoniaei,* JVLP-A-1-N211; (**M**) *Cibotiumspora jurienensis,* JVLP-3-2-L240; (**N**) *Todisporites minor*, JVLP-3-2-F502; (**O**) *Baculatisporites comaumensis*, JVLP-1-2-H083; (**P**) *Apiculatisporites* sp., JVLP-4-1-L222; (**Q**) *Dictyophyllidites, mortonii,* JVLP-2-4-K370; (**R**) *Nevesisporites vallatus*, JVLP-3-1-G221; (**S**) Spore indeterminate, JVLP-B-2-F070; (**T**) *Obtusisporites* sp., JVLP-A-3-O431.

**Figure 6:**
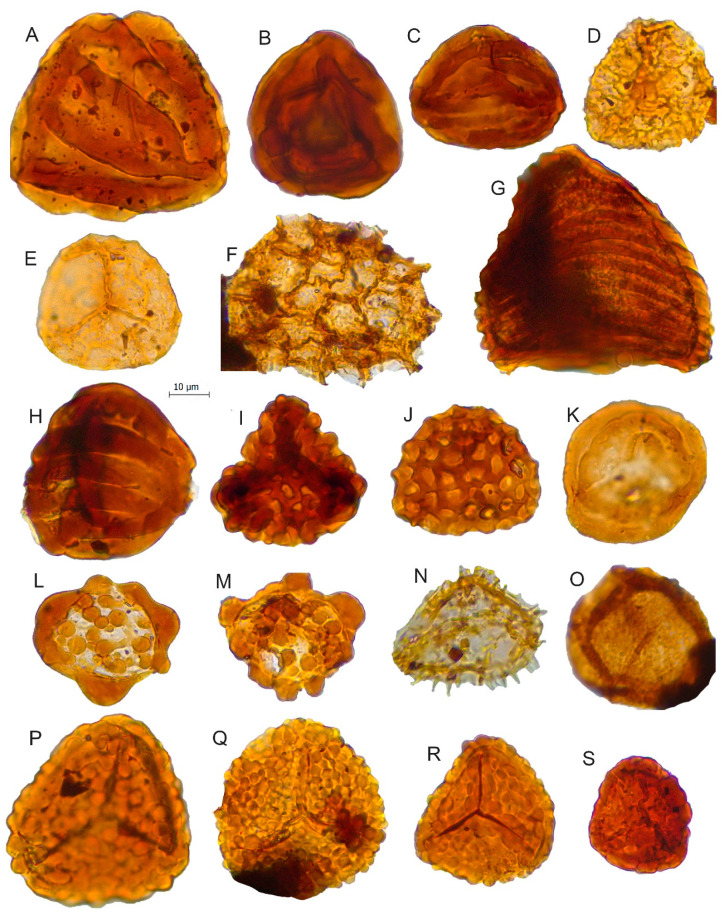
Scale bar: 10 µm. (**A**) *Striatella balmei*, JVLP-2-2-V421*; (***B**) *Striatella seebergensis*, JVLP-1-1-X413*; (***C**) *Striatella cooksoniae*, JVLP-3-2-F332*; (***D**) *Retitriletes pseudoreticulatus*, JVLP-B-2-M422*;* (**E**) *Retitriletes* sp., JVLP-2-4-M252; (**F**) cf. *Retitriletes* sp*.,* JVLP-2-4-U373; (**G**) *Contignisporites globulentus*, JVLP-1-2-N340; (**H**) *Contignisporites fornicatus*, JVLP-3-2-M503; (**I**) *Ischyosporites marburgensis*, JVLP-2-2-B220; (**J**) *Klukisporites lacunus*, JVLP-3-2-K531; (**K**) *Polycingulatisporites crenelatus,* JVLP-3-1-W300; (**L**) *Patellasporites distaverrucosus*, JCLP-B-4-K402; (**M**) *Patellasporites* cf. *distaverrucosus,* JVLP-3-2-M380; (**N**) *Neoraistriskia* sp. JVLP-B-1-S420; (**O**) *Osmundacidites wellmanii,* JCLP-C-1-L400; (**P**) *Leptolepidites verrucatus,* JVLP-3-2-J261; (**Q**) *Leptolepidites major*, JVLP-B-2-H393; (**R**) *Leptolepidites* sp., JVLP-3-1-k410; (**S**) *Leptolepidites crassibalteus*, JVLP-C-1-G340.

**Figure 7:**
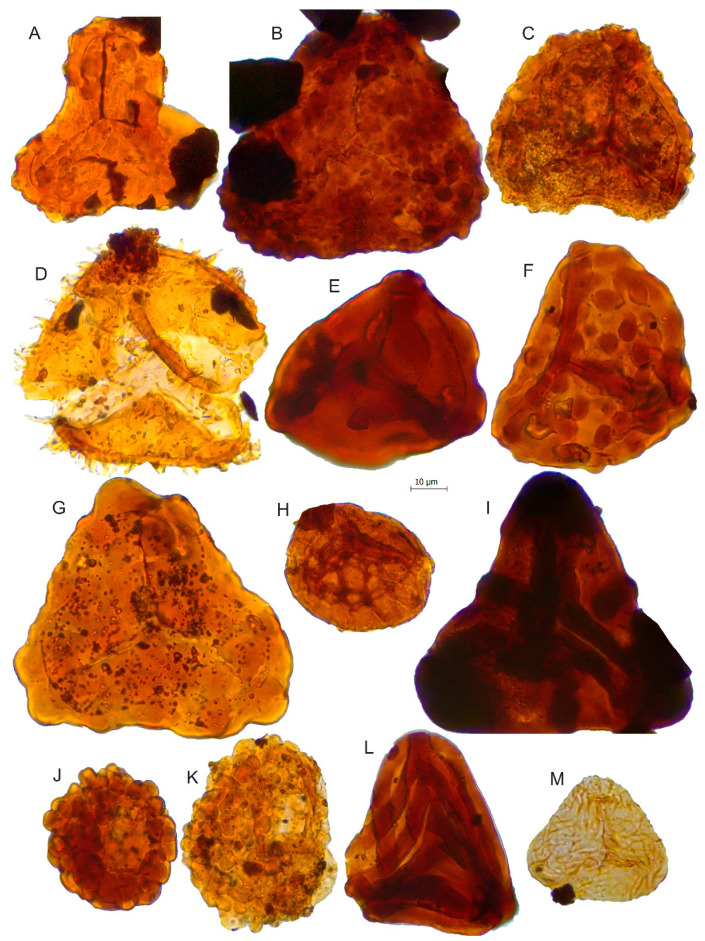
Scale bar: 10 µm. (**A**) *Impardecispora apiverrucata*, JVLP-A-1-C114; (**B**) *Impardecispora* sp., JCLP-B-4-R260; (**C**) *Concavissimisporites variverrucatus*, JVLP-3-1-f464; (**D**) *Pilosisporites trichopapillosus*, JVLP-B-2-P401; (**E**) *Trilobosporites* sp.1, JVLP-3-1-H190; (**F**) *Concavissimisporites montuosus*, JVLP-3-2-J390; (**G**) *Trilobosporites* cf. *canadensis*, JVLP-1-2-L272; (**H**) *Triporoletes* sp.?; (**I**) *Trilobosporites* sp.2, JCLP-C-2-K350; (**J**) *Cerebropollenites macroverrucosus*, JVLP-3-2-G420; (**K**) *Cerebropollenites* cf. *mesozoicus*, JVLP-2-4-O340; (**L**) *Cicatricosisporites* cf. *pseudotripartitus*, JVLP-3-1-S331; (**M**) *Cicatricosisporites sinuosus*, JVLP-A-2-P051.

**Figure 8:**
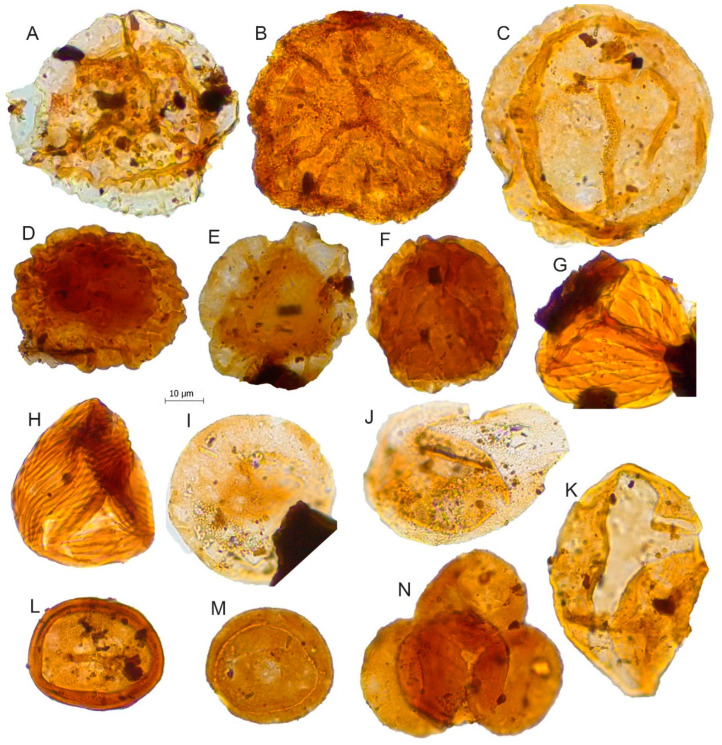
Scale bar: 10 µm. (**A**) *Aequitriradites spinulosus*, JVLP-A-M260; (**B**) *Densoisporites velatus*, JVLP-A-EX-G271; (**C**) *Araucariacites australis*, JVLP-1-2-J494; (**D**) *Callialasporites segmentatus*, JCLP-E-1-U120; (**E**) *Callialasporites trilobatus*, JVLP-3-1-V240; (**F**) *Callialasporites microvelatus*, JVLP-3-1-K494; (**G**) *Ruffordiaspora* sp.1, JVLP-A-EX-Q121; (**H**) *Ruffordiaspora australiensis*, JCLP-C-1-D280; (**I**) *Exesipollenites tumulus*, JVLP-3-1-G391; (**J**) *Perinopollenites elatoides*, JVLP-3-1-G490; (**K**) *Cycadopites follicularis,* JVLP-2-4-K310; (**L**) *Classopollis classoides*, JCLP-E-2-N254; (**M**) *Classopollis simplex*, JVLP-3-2-D421; (**N**) Tetrad of *Classopollis* sp., JVLP-2-4-E210.

**Figure 9:**
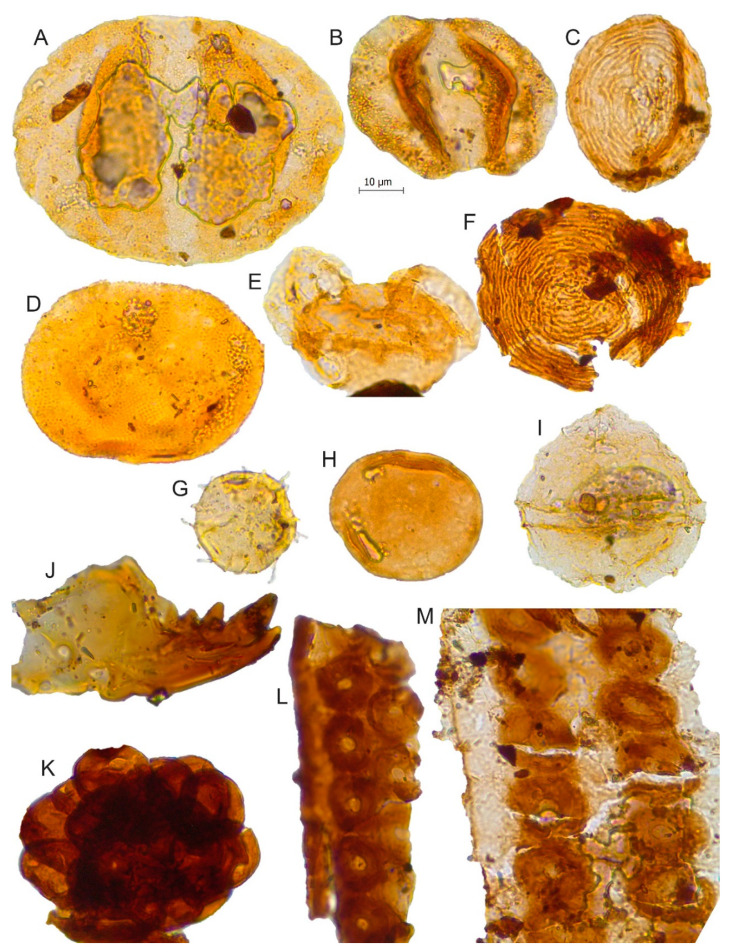
Scale bar: 10 µm. (**A**) *Alisporites grandis,* JVLP-3-1-Q240; (**B**) *Alisporites* cf. *similis,* JVLP-3-1-D132; (**C**) *Chomotriletes minor,* JVLP-2-2-J132; (**D**) *Tasmanites* sp., JVLP-3-1-E460; (**E**) *Pinuspollenites* sp., JVLP-2-4-T262; (**F**) *Chomotriletes fragilis,* JVLP-A-1-L272; (**G**) *Michrystridium* sp.1, JVLP-1-1-F490; (**H**) *Spheripollenites* sp. JVLP-2-2-C352; (**I**) Gonyaulacaceae gen. et sp. indet., JVLP-3-2-M501; (**J**) Scolecodont (annelid jaw), JCLP-C-1-D370; (**K**) Cluster of spores*,* JVLP-C-2-F490; (**L**) Tracheid: Biseriate alternate pits (*Araucarioxylon*?), JCLP-E-1-C260; (**M**) Tracheid: Biseriate pits, JCLP-E-1-G431.

The samples were analysed using standard palynological techniques [37] at the Palynology Laboratory of the Department of Marine Geosciences at the University of Vigo. Briefly, 10–15 g of material was crushed and treated with hydrochloric (HCl 10%) and hydrofluoric acid (HF 70%) in order to dissolve carbonates and remove silicate components, followed by application of hot 10% HCl to dissolve silica gel formed during HF treatment. The residue was sieved using a nylon filter with a mesh of 10 µm; the organic residue was washed with distilled water and sprayed on a coverslip using cellosize (hydroxyethyl cellulose), dried, and mounted on a microscope slide. For the SEM observation (Figure 4), the organic residue was dried and covered with gold. The palynological slides were observed and photographed under a Leica ICC50W (Leica Camera, Wetzlar, Alemania) optical microscope at 1000× magnification (Figure 5, Figure 6, Figure 7, Figure 8 and Figure 9). Some palynomorphs and small wood remains present in the slides were re-examined under the Scanning Electron Microscope (SEM) JEOL JSM6010LA (JEOL Ldt., Tokyo, Japan) at CACTI (Centro de Apoio Científico-Tecnolóxico á Investigación, University of Vigo). The position of each illustrated palynomorph is given in the figure captions according to the “England Finder” graticule.

The relative dominance of the palynomorphs was analysed as an entire unity for all the samples because of the lack of sedimentary rate continuity and the frequent reworked levels in these sections. The palynomorph dominance data in this work, should be understood as qualitative more than quantitative.

The botanical affinities of the palynomorphs found in the Lastres Fm. are shown in the Table 1 after a selection of references where the relation between the palynomorph and its producer is indicated.

## 4. Results

All the palynological samples yielded palynomorphs, showing a high diversity with different levels of preservation. The samples taken from Colunga municipality (JCLP) presented an acceptable richness and preservation, while the samples collected in the Villaviciosa municipality (JVLP) presented, in general, greater richness and preservation due to taphonomical processes. A total of 62 different (morpho)species were identified, belonging to at least 49 (morpho)genera (see Table 1). The different stratigraphic levels showed very similar compositions among them. This fact, in addition to the complexity to correlate the different sections of the Lastres Fm. based on its sedimentology, led us to consider all the studied palynological samples as a single assemblage.

The diversity of this palynoflora present sporo-pollen dominance, where the dominant groups are the spores of pteridophytes and bryophytes (representing half of the identified palynomorphs), while the other half corresponded to pollen from different groups of gymnosperms. Less than 5% of palynomorphs are non-pollen-palynomorphs (NPPs), including dinoflagellates, algae, and fungi spores. During the palynological analysis, different wood fragments, unidentifiable cuticle remains, and phytoclasts with different grades of opacity were also found.

The relative abundance of the taphonomy in this assemblage is dominated by *Spheripollenites psilatus*, which is usually linked to conifers. The second most abundant palynomorph was *Leptolepidites* followed by bisaccate pollen of the genus *Alisporites*, pollen from *Classopollis* spp., and trilete spores of *Cyathidites* spp. The other genera identified had a minor representation of relative abundance.

## 5. Discussion

### 5.1. Palynostratigraphical Implications

Most of the taxa present in the Lastres Fm. have a wide biostratigraphic range, and many of the genera found are very common and representative of palynological assemblages from the Upper Jurassic and Lower Cretaceous of Europe, such as *Leptolepidites*, *Callialasporites*, *Classopollis*, *Concavissimisporites*, *Retitriletes*, *Striatella*, *Cyathidites*, *Contignisporites*, or *Dyctiophyllidites*. Nevertheless, some of the genera, such as *Cicatricosisportes*, *Ruffordiaspora*, *Pilosisporites*, or *Aequitriradites,* have species with a more restricted range.

The first occurrences of *Cicatricosisporites*-*Ruffordiaspora* complex in the fossil record occur in the Upper Jurassic. Dettmann and Clifford [46] suggested that their first records could take place in the Oxfordian or even in the Callovian. However, the first clear appearances of *Cicatricosisporites* in Europe and Africa occur in the Kimmeridgian of Norway [64], Ethiopia [65], and Egypt [66]. The first occurrence of *Cicatricosisporites* in the Iberian Peninsula has been placed in Lower Kimmeridgian deposits from Segura de la Sierra in Jaen province, Spain [31].

The *Cicatricosisporites*-*Ruffordiaspora* morphospecies present in this palynological assemblage are *C*. cf. *pseudotripartitus*, *C*. *sinuosus*, and *R*. (*C*.) *australiensis*. The older occurrences of *Ruffordiaspora australiensis* were found in the Kimmeridgian of North America [67,68], the North Atlantic offshore [69], Egypt [70], and other parts of Europe [71,72]. In the Iberian Peninsula, it is also known from the Kimmeridgian deposits of Portugal [73]. The first occurrence of *Cicatricosisporites pseudotripartitus* is in the Kimmeridgian of Egypt [66] while, in the Iberian Peninsula, it is only found in Cretaceous deposits, more specifically in the Berriasian of the Cameros Basin in Northern Spain [33]. However, although the distal face of the specimen from the Lastres Formation (see Figure 7L) is compatible with *C*. *pseudotripartitus*, the proximal face is not observable, so we cannot be sure if it corresponds to this species, therefore we prefer to be cautious with its biostratigraphic utility. The presence of *Cicatricosisporites sinuosus* is a bit more problematic in the Upper Jurassic Lastres Formation since its presence is more common in the Cretaceous [74]. Currently, its first occurrence is in the Purbeck Group (Tithonian-Berriasian) of England, where the holotype of this species was found [75], so this record from Asturias would be the first record of this taxon in pre-Tithonian deposits.

The first appearance of *Aequitriradites spinulosus* was in the Upper Jurassic, and in Europe, the oldest record was found in the Tithonian of Romania [76]. However, in the Iberian Peninsula this taxon was only recorded in Cretaceous deposits, having its first occurrence in this region in the lowermost Berriasian of the Galve sub-Basin in the Teruel province [32] and the Early Berriasian of the Cameros Basin in Burgos province [33]. It was also frequently found along the Early Cretaceous in the Valanginian-Barremian of Burgos province [77], the Lower Valanginian of the NW Iberian offshore [78], the Barremian from Maestrazgo Basin [79] and Peñacerrada in Alava province [80], and also in the Hauterivian, Barremian and Aptian from Portugal [81,82]. Consequently, the presence of *A. spinulosus* in the Kimmeridgian strata of the Lastres Formation represents the oldest record of this species in Europe.

The oldest record of *Impardecispora apiverrucata* apparently occurs in the Bathonian of Iran (see plate 1, Figure 8 in [83]), nevertheless the quality of the illustration provided by these authors does not allow to check the correct identification of this palynomorph. Anyway, the first appearance in the Iberian Peninsula is from the Tithonian-Berriasian deposits of Porto Pinheiro and Vale Painho in Portugal [29,84] and in the Berriasian of the Cameros Basin in Spain [33]. In Europe, the presence of this morphospecie in pre-Tithonian deposits is rare, and its presence in the Lastres Fm. constitutes the oldest record in the Iberian Peninsula.

The genus *Patellasporites* is relatively common in the Cretaceous of the Iberian Peninsula [79,84,85], while the oldest record of this genus in this region comes from the Tithonian deposits of Portugal [28]. Therefore, the presence of *P*. *distaverrucosus* in the studied levels represents the oldest record of this genus in the Iberian Peninsula and the first Jurassic record for this morphospecies worldwide.

The oldest record of *Pilosisporites trichopapillosus* comes from the Upper Kimmeridgian of Canada [86,87], but this species only becomes common from the Berriasian onwards. In northern Europe, the oldest evidence of this species is in the Berriasian [71,88], and in the Iberian Peninsula its first occurrence was found in Vale Painho, Portugal [84,85].

Previous studies have indicated that the age of the Lastres Fm. is clearly Upper Jurassic, and the formation was assigned to the late Lower Kimmeridgian (Cymodoce chronozone) by Olóriz et al. [6] based on the presence of some ammonites. On the other hand, the middle part of the Lastres Fm. was referred to the Upper Kimmeridgian (Eudoxus chronozone), also with ammonites by Dubar and Mouterde [15] and Suárez-Vega [14]. Otherwise, Ramírez del Pozo [17] suggests a Portlandian (=late Tithonian) age for the upper part of this formation. Moreover, recent interpretations also suggest a late Kimmeridgian age for the lower and middle part of the formation and an early Tithonian age for its upper part based on the ostracod assemblages [7]. Additionally, González-Fernández et al. [5], based on correlation of sedimentary sequences, suggested a Kimmeridgian-early Tithonian age for the lower and middle part of the Lastres Fm. and a Tithonian age for the upper part, and the presence of *Protocupressinoxylon purbeckensis* in the Tereñes and Lastres formations is in tune with the Kimmeridgian-Tithonian age for these strata [18].

Most of the taxa found in the palynological assemblage of the Lastres Fm. deposits present a wide stratigraphic range that encompasses much of the Jurassic and Cretaceous periods. However, no evidence was found to suggest a Cretaceous age. The eventual presence of some taxa such as *Cicatricosisporites* cf. *pseudotripartitus*, *C*. *sinuosus*, *R*. (*C*.) *australiensis*, *Aequitriradites spinulosus*, *Impardecispora apiverrucata*, *Patellasporites distaverrucosus*, and *Pilosisporites trichopapillosus*, are not compatible with pre-Kimmeridgian deposits, having affinities with the classical Tithonian palynofloras (or relatively close to the Jurassic-Cretaceous boundary). However, the presence of ammonites in one of the studied sections [6] shows that the age of the lower part of the Lastres Fm., is probably late Early Kimmeridgian even though our palynological assemblage presents more Tithonian than Kimmeridgian affinities. The Lastres Fm. extends for tens of kilometres along the Asturian coast, so further palynological and biostratigraphic studies are necessary in new sections of this formation in order to make a more detailed interpretation of its age range.

### 5.2. Palaeoenvironment and Palaeoecology

This palynological assemblage from the Lastres Formation is mainly dominated by palynomorphs of continental origin (spores fundamentally). However, prasinophytes, dinoflagellate cysts, and scolecodonts probably of autochthonous origin, have also been found indicating that the depositional setting was a transitional environment with marine influence like a paralic sedimentary environment. This interpretation is consistent with a deltaic environment previously reported for the Lastres Fm. by other authors [3,36] that is in tune with the occasional presence of remains of open marine ammonites in the studied sections [6]. The possibility of an open marine depositional setting close to the coast is not consistent with this palynological assemblage, since palynological slides are plenty of well-preserved cuticles and wood remains with little transport. In addition, the presence of tree trunks preserved in situ in life position and also the presence of different remains of macroflora [2] reinforce this interpretation of a transitional (paralic) environment.

The quality of preservation of the palynomorphs is not homogeneous. This could be explained because some of them probably suffer long-distance transport representing allochthonous pollen/spores, which is the case with some poorly preserved taxa such as *Pilosisporites* and *Aequitriradites*, with affinities related to ferns and mosses, respectively [40]. These two groups of plants are less tolerant to salinity, living in more protected and continental environments. On the other hand, most of the bisaccate pollen grains present a deficient to bad preservation and they could be allochthonous in origin. Abbink [53] and Abbink et al. [52] argue that most of the bisaccate pollen (such as *Alisporites*) could have their origin in upland SEGs (sporomorph eco-group) which is consistent with previous interpretation since these upland areas would be located several kilometres away from the depositional setting.

On the other hand, in addition to the parautochthonous or allochthonous prasinophytes, dinoflagellate cysts, scolecodonts, and foraminiferal test lining, the presence of unseparated tetrads of *Classopollis* sp. is suggestive of a little transport from the parent-plant that is likely representative of the autochthonous flora. *Classopollis*, is a pollen commonly related to tree-size conifers belonging to the Cheirolepidiaceae family, that show xerophytic and halophytic adaptations inhabiting disturbed environments as they are coastal areas [52,55]. In particular, Volkheimer et al. [60] considered *Classopollis* from the Lajas Formation in the Middle Jurassic of the Neuquén Basin (Argentina) as a thermophilic coastal proxy related to extraordinary flooding in well-drained soils of the delta plain.

**Figure 10:**
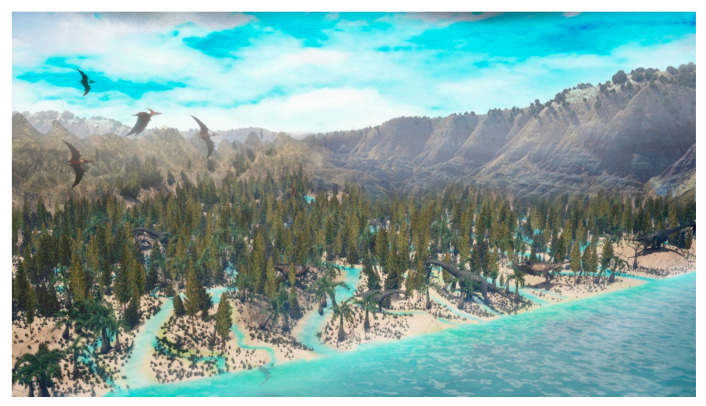
Palaeoreconstruction of “The Dinosaur Coast” in the Kimmerdigian-Tithonian of Asturias, taking into account the new palaeobotanical data about the Lastres Fm. (Artist: Jose Luis Suso).

The mixture of taxa related to different botanical affinities and associated with different types of environments, such as marine-influenced brackish environments (dinoflagellate cyst, foraminiferal test linings, prasinophytes, etc.), coastal (*Classopollis* spp. was one of the more abundant palynomorphs with *Araucariacites*, as well as *Callialasporites*; *Exesipollenites* are also present), high-sinuosity rivers/lowlands (most of the fern and bryophyte spores; see Table 1) and uplands (*Alisporites* was the third most common genera in the Lastres assemblage) suggests that during the Late Jurassic the Asturian coast dominated by deltas would be composed of different subenvironments each of them dominated by different botanical communities. Taking into account previous sedimentological data and observations during the collection of the palynological samples, we have drawn an interpretation of the most probable distribution of the plant communities in the Asturian environments of the Upper Jurassic, based on the classification in SEGs (Sporomorphs Eco-groups; *sensu* [52,53]) of the different palynomorphs (Figure 10).

On the one hand, there are a group of palynomorphs that would be related to upland eco-groups, as would be the case of the bisaccate pollen such as *Alisporites* (the third most frequent genus in the assemblage) or *Pinuspollenites*. These palynomorphs present worse preservation than other taxa which could indicate an allochthonous origin, which would be consistent with upland areas [52,53] that were further away from the depositional setting. Most of these groups of bisaccate forms have been related to conifers of families Podocarpaceae and Pinaceae [41], which would inhabit areas of higher altitude.

Many of the taxa present in the Lastres Fm. show affinities with lowland SEG (e.g., *Cicatricosisporites*, *Concavissimisporites*, *Cycadopytes*, *Contignisporites*, *Deltoidosora*, *Striatella, Gleicheniidites*, *Impardecispora*, *Ischyosporites*, *Matonisporites*, *Monosulcites*, *Osmundacidites*, *Perinopollenites*, or *Trilobosporites*). These lowland communities probably would be vegetation close to freshwater swamps and ponds in flood plains, with combination of taxa adapted to drier and wetter environmental conditions [52,53], likely without influence of salt water. In the case of the Lastres Fm., these plant communities would be placed in the lower delta plain, which is an area with little marine influence dominated by swampy conditions and areas of freshwater. Most types of ferns need shady and humid environments to grow, and hence they would be located closer to the freshwater masses. However, other taxa of this SEG could be located in less humid areas of the lowland. For example, the ferns of the Family Matoniaceae (represented in this assemblage by both form-genera *Dictyophyllidites* and *Matonisporites*) currently inhabit the mountain slopes in the Malaysian Archipelago and present cuticles that make them resistant to differences of temperature during the day [89,90]. Nevertheless, the distribution of this family of ferns during the Mesozoic was wider and its possible habitats were more diverse [90]. It is also the case of the ferns of the family Gleicheniaceae, represented in the Lastres Fm. by the genus *Gleicheniidites*, which are resistant to direct sunlight. These ferns are currently common in subtropical regions [89]. Although most of the taxa of this type of plant community would correspond to pteridophytes (see botanical affinities in Table 1), woody plants from other groups such as Cycadophytes [41] or taxodiaceous conifers would also be common in Late Jurassic communities [52,91].

Some species related to river SEG would be placed associated with freshwater fluvial channels [52,53], such as *Leptolepidites* spp. (the second most abundant taxon), or *Stereisporites* and *Staplinisporites*, which are related to Bryophytes and Lycopodiaceae-Selaginellaceae [41]. These plant groups present high water requirements consistent with riverbank communities that offered constant humidity and were possibly periodically submerged [52,53].

Finally, in the Lastres Fm., the palynomorphs related to coastal environments are abundant (Coastal SEG), corresponding to pollen or spore plant producers that would grow close to the coast possibly supporting conditions of certain salinity. Taking into account the sedimentology of the study area, these plant communities were possibly located in the delta plain and the palynomorphs related to this SEG would be *Classopollis* (=*Corollina*), *Callialasporites*, *Araucariacites*, and *Exesipollenites* [52,53]. *Exesipollenites* presents affinities with the family Cupressaceae and both *Callialasporites* and *Araucariacites* have been related to Araucaraceae conifers [65,66,69,73]. *Classopollis* was one of the most abundant pollen grains, clearly related to Cheirolepidiaceae [63]. This is a group of abundant and diversified conifers in the Mesozoic, many of which would be adapted to coastal conditions and with tendency to establish extensive forests in tropical to subtropical lowland areas [63]. Although their ecological affinities are controversial, they have been widely related to thermophilus and drought-resistant shrubs and trees [52,53]. In the Lastres Fm., Cheirolepidiaceae was already known due to the presence of wood remains attributed to *Protocupressinoxylon purbeckensis* [18,92], a tree-like taxon (about 13 m in altitude) that has also been associated with coastal ecosystems and with a certain tolerance to salinity (spray or salty soils) [18,93].

On the other hand, the most abundant palynomorph, *Spheripollenites,* presents an ambiguous biological affinity [94,95], frequently assigned to gymnosperm pollen. It was related to the inner bodies of Cheirolepidiaceae-Cupressaceae pollen [52,53,96] while some authors have associated it with other families such as Cupressaceae [97] or Araucariaceae [74]. The presence of a large number of *Spherinopollenites* spp., as occurs in the Lastres Fm. together with a large number of *Classopollis*, was related in some cases to a more developed annual dry season [29,52,53,63,98]. The interpretation of a seasonal climate during the Kimmeridgian in the Asturias area is also supported by the presence of several palynomorphs linked with different climate and environmental conditions. These climatic interpretations are partially consistent with previous data for the underlying Lower Kimmeridgian Vega Formation based on palaeosoil studies. The sediments of Vega Fm. were deposited during a subhumid to semiarid seasonal climate [99], and these conditions are also compatible with the palynological assemblage found in the Lastres Fm. At the same time, this is in tune with the palaeoecological conditions observed for the Upper Jurassic of other regions in Northern Spain according to the palaeobotanical macro remains [100]. In addition, similarities in the fauna assemblages [99,101] and in the palaeoenvironmental conditions between the Upper Jurassic formations from Asturias and the Morrison Fm. (western North America) have been found [99]. In the Morrison Fm. and in the Vega Fm., a seasonally variable precipitation regime was interpretated, although the Morrison Formation would represent a drier and warmer environment [99].

Moreover, the abundant tracks and remains of giant plant-eating dinosaurs such as sauropods, stegosaurs, and ornithopods in the studied strata of the Lastres Fm. [3,13,101] suggest the presence of abundant and diverse vegetation. Therefore, the denser vegetation areas (possibly the plant communities associated with river channels and ponds or swamps in the most protected area of the delta; see Figure 10) served as food sources for these groups of dinosaurs.

### 5.3. Evidence of Wildfires

The detailed analysis of the samples in the scanning electron microscope (SEM) allowed us to identify the frequent presence of charcoalified wood remains. Small fragments showing homogenised cell walls (see Figure 4M,N) occur when the wood is exposed to the fire. Some of the remains are composed of secondary xylem with wood cells that present both empty cell lumina and walls with homogenised middle lamellas (Figure 4M) indicating high temperatures during the combustion process ranging from 220–230 °C [102] to 300–325 °C [103] depending on the different studies and the possible oxygen ingress on the fire system [104]. Nevertheless, some of the charcoalified remains still preserve a thin separation between lamellas (Figure 4M) while other records show middle lamellas that are not fused (Figure 4O) indicating a variety of regimes of wildfires in the Kimmeridgian palaeoenvironments of Asturias during the deposition of the Lastres Formation. In addition, the presence of these charcoalified remains in all the studies samples suggests that the wildfires were recurrent in this formation.

## 6. Conclusions

The studied sections from the Lastres Fm. in northwestern Spain reveal a very homogeneous palynofloral assemblage, in terms of diversity, along the different stratigraphical levels. A total of 62 morphospecies and 49 morphogenera of palynomorphs have been identified, including pteridophyte and bryophyte spores, gymnosperm pollen, acritarchs, dinoflagellate cysts, marine, and freshwater algae, scolecodonts, as well as cuticle and wood debris. The relative dominance of continental palynomorphs and the presence of some dinoflagellate cysts, Prasinophyceae, and scolecodonts suggest a transitional depositional setting, with an occasional marine influence, but with indicators of freshwater, compatible with interdistributary depositional environments. The age of some key taxa indicates that the palynological assemblage cannot be older than the Kimmeridgian, indicating a Kimmeridgian-Tithonian age. The botanical and environmental affinities of the pollen and spores suggest the presence of different vegetation, including plant communities in humid areas such as riverbanks (riparian) and small freshwater ponds (dominated by bryophytes and ferns) and a coastal plant community that would inhabit more arid areas (dominated by gymnosperms and some pteridophytes), where these masses of vegetation probably offered food and protection to some of the herbivorous dinosaurs of the Lastres Fm. The mixed climatic preferences of the studied taxa suggest a seasonal environment for the Kimmeridgian of Asturias, in tune with previous palaeontological and sedimentological studies. In addition, the dominance of some palynomorphs is indicative of arid and warm conditions at least during some periods or seasons. Finally, the SEM analyses of microscopical wood remains indicate the presence of wildfires during the Kimmeridgian in “The Dinosaur Coast” affecting the plant communities in this zone.

## Figures and Tables

**Table 1:** Palynomorphs identified in the Lastres Fm., with their botanical affinities.

Taxa	Figure	Botanical Affinity (Reference)
**Spores**		
*Aequitriradites spinulosus*	Figure 8A	Bryophytes, Hepaticeae [38,39,40]
*Apiculatisporites* sp.	Figure 5P	Lycopodiaceae/Selaginellaceae-type [41,42,43]
*Baculatisporites comaumensis*	Figure 5O	Osmundaceae-type [41]
*Biretisporites potoniaei*	Figure 5L	Schizaeaceae [44,45]
*Camarazonosporites rudis*	Figure 4F and Figure 5H	Lycopodiaceae [41,42]
*Cibotiumspora jurienensis*	Figure 5M	Cyatheaceae/Dicksoniaceae [41,42]
*Cicatricosisporites* sp.	Figure 4A	Schizaeaceae [41,46]
*Cicatricosisporites pseudotripartitus*	Figure 7L	
*Cicatricosisporites sinuosus*	Figure 7M	
*Concavissimisporites montuosus*	Figure 7F	Dicksoniaceae/Cyatheaceae [47]
*Concavissimisporites variverrucatus*	Figure 7C	
*Contignisporites cooksoni*	Figure 4C	Schizaeaceae [41,48]
*Contignisporites fornicatus*	Figure 6H	
*Contignisporites globulentus*	Figure 6G	
*Cyathidites australis*	Figure 5A	Dicksoniaceae, Cyatheaceae, Dipteridaceae, Matoniaceae [41,49,50]
*Cyathidites minor*	Figure 5B	
*Deltoidospora* sp.	Figure 5D	Cyatheaceae [51,52]
*Dictyophyllidites harrisii*	Figure 4K	Dipteridaceae/Matoniaceae [41]
*Dictyophyllidites mortonii*	Figure 5Q	
*Gleicheniidites senonicus*	Figure 5C	Gleicheniaceae [41]
*Impardecispora apiverrucata*	Figure 7A	Cyatheaceae [52]
*Impardecispora* sp.	Figure 7B	
*Ischyosporites marburgensis*	Figure 6I	Schizaeaceae [41]
*Klukisporites lacunus*	Figure 6J	
*Klukisporites* sp.	Figure 4D	
*Leptolepidites crassibalteus*	Figure 6S	Lycopodiaceae/Selaginellaceae [41,52,53]
*Leptolepidites major*	Figure 6Q	
*Leptolepidites* sp.	Figure 4B and Figure 6R	
*Leptolepidites verucatus*	Figure 6P	
*Matonisporites equiexinus*	Figure 5F	Dipteridaceae/Matoniaceae [41]
*Matonisporites* sp.	Figure 5G	
*Murospora* sp.	Figure 5E	
*Neoraistriskia* sp.	Figure 6N	
*Densoisporites velatus*	Figure 8B	
*Nevesisporites vallatus*	Figure 5R	
*Obtusisporis canadansis*	Figure 5T	Abortive spores [41]
*Osmundacidites wellmanii*	Figure 6O	Osmundaceae [41]
*Patellasporites distaverrucosus*	Figure 6L,M	Selaginellaceae [54]
*Pilosisporites trichopapillosus*	Figure 7D	Pteridophytes [40]
*Polycingulatisporites crenelatus*	Figure 6K	Bryophytic Spores Sphagnaceae-type [41]
*Retitriletes* sp.	Figure 6F	Bryophyte (Lycopodiaceae) [52,55,56]
*Retitriletes pseudoreticulatus*	Figure 6D	
*Retitriletes clavatoides*	Figure 4G	
*Retitriletes* sp.	Figure 6E	
*Ruffordiaspora australiensis*	Figure 8H	Schizaeaceae Ruffordia-type [46]
*Ruffordiaspora* sp.1	Figure 8G	
*Staplinisporites caminus*	Figure 5I	Lycopodiaceae [41]
*Staplinisporites* sp.	Figure 5J	
*Stereisporites antiquasporites*	Figure 5K	Bryophyte, Sphagnaceae [41]
*Striatella balmei*	Figure 4H and Figure 6A	Pteridaceae [57]
*Striatella cooksoniae*	Figure 6C	
*Striatella scanica*	Figure 4I	
*Striatella seebergensis*	Figure 6B	
*Todisporites minor*	Figure 5N	Osmundaceae [41]
*Trilobosporites canadensis*	Figure 7G	Schizaeaceae [41]
*Trilobosporites* sp.1	Figure 7E	
*Trilobosporites* sp.2	Figure 7I	
*Triporoletes* sp.	Figure 7H	Bryophyte [58]
**Pollen**		
*Alisporites* cf. *similis*	Figure 9B	Pinaceae/Podocarpaceae [41,52,53]
*Alisporites grandis*	Figure 9A	Pinaceae/Podocarpaceae [41,52,53]
*Araucariacites australis*	Figure 8C	Araucariaceae [41,59,60]
*Callialasporites dampieri*	Figure 4E	
*Callialasporites microvelatus*	Figure 8F	
*Callialasporites segmentatus*	Figure 8D	
*Callialasporites trilobatus*	Figure 8E	
*Cerebropollenites* cf. *mesozoicus*	Figure 7K	Sciadopityaceae/Cupressaceae (former Taxodiaceae) [52,53,61,62]
*Cerebropollenites macroverrucosus*	Figure 7J	
*Classopollis classoides*	Figure 8L	Cheirolepidiaceae [52,53,61]
*Classopollis simplex*	Figure 8M	
*Classopollis*	Figure 8N	
*Cycadopites follicularis*	Figure 8K	Cycadopsida/Pteridospermopsida [41]
*Exesipollenites tumulus*	Figure 8I	Bennettitales [52,53]
*Perinopollenites elatoides*	Figure 8J	Cupressaceae (former Taxodiaceae) [52,63]
*Pinuspollenites* sp.	Figure 4L and Figure 9E	Pinaceae/Podocarpaceae [41]
*Spheripollenites* sp.	Figure 9H	Cupressaceae (former Taxodiaceae)/Cheirolepidiaceae [52,53]
**Others (Dinoflagellate, Achritarchs, cuticle…)**		
*Botryococcus* sp.	Figure 4J	Algae
*Chomotriletes fragilis*	Figure 9F	
*Chomotriletes minor*	Figure 9C	
Wood remains	Figure 4M,N	Gymnosperms
Cuticle remains	Figure 4O	Plantae
Gonyaulacaceae gen. et sp. indet.	Figure 9I	Dinoflagellates
*Michrystridium* sp.1	Figure 9G	Acamhomorphitae
Scolecodont	Figure 9J	Annelid
*Tasmanites* sp.	Figure 9D	Tasmanititae (prasinophytes)
Tracheid type 1	Figure 9L	Gymnosperms (Araucariaceae?)
Tracheid type 2	Figure 9M	Gymnosperms

## Data Availability

The palynological slides are stored in the Department of Marine Geosciences of the Vigo University (Spain).
